# Mast Cell Functions Linking Innate Sensing to Adaptive Immunity

**DOI:** 10.3390/cells9122538

**Published:** 2020-11-25

**Authors:** Konstantinos Katsoulis-Dimitriou, Johanna Kotrba, Martin Voss, Jan Dudeck, Anne Dudeck

**Affiliations:** 1 Institute for Molecular and Clinical Immunology, Otto-von-Guericke Universität Magdeburg, 39120 Magdeburg, Germany; konstantinos.katsoulis-dimitriou@med.ovgu.de (K.K.-D.); johanna.kotrba@med.ovgu.de (J.K.); martin.voss@med.ovgu.de (M.V.); jan.dudeck@med.ovgu.de (J.D.); 2 Health Campus Immunology, Infectiology and Inflammation, Medical Faculty, Otto-von-Guericke Universität Magdeburg, 39120 Magdeburg, Germany

**Keywords:** mast cell, adaptive immunity, dendritic cell, T cell, antigen presentation

## Abstract

Although mast cells (MCs) are known as key drivers of type I allergic reactions, there is increasing evidence for their critical role in host defense. MCs not only play an important role in initiating innate immune responses, but also influence the onset, kinetics, and amplitude of the adaptive arm of immunity or fine-tune the mode of the adaptive reaction. Intriguingly, MCs have been shown to affect T-cell activation by direct interaction or indirectly, by modifying the properties of antigen-presenting cells, and can even modulate lymph node-borne adaptive responses remotely from the periphery. In this review, we provide a summary of recent findings that explain how MCs act as a link between the innate and adaptive immunity, all the way from sensing inflammatory insult to orchestrating the final outcome of the immune response.

## 1. Introduction

Mast cells (MCs) are well-known as key effector cells of type I allergic reactions, commonly named anaphylactic responses. In this case, MCs are activated by the crosslinking of cell-surface-bound FcεRI-IgE complexes by a specific antigen, which results in a three-step-response: (a) The immediate degranulation of MC secretory granules; (b) the release of lipid mediators (including thromboxanes, prostaglandins, and leukotrienes); and (c) the secretion of a wide spectrum of de novo synthesized mediators (including cytokines, chemokines, and growth factors) [1,2,3]. However, MCs are also equipped with a spectrum of surface receptors allowing the sensing of various pathogen-associated patterns (PAMPS), danger-associated molecular patterns (DAMPS), cytokines, chemokines, neuropeptides, and others [1,3,4,5,6,7]. Moreover, the mode of ligand-receptor-based MC activation and downstream signaling determines the mode of MC action, which can consist of the full three-step-response, but can also solely involve de novo synthesized mediator release, without degranulation [6,8]. Based on this spectrum of sensing capacities, and the ability to deploy specific responses, there is increasing evidence that MCs critically contribute to innate host defense against pathogens. Additionally, MCs influence the induction, amplitude, and function of the adaptive arm of the immune defense, either by direct effects on T cells or indirectly, by modifying the properties of antigen-presenting cells (APCs) [6,9]. Importantly, MCs even modulate lymph node-borne adaptive responses remotely from the periphery. In this review, we provide a summary of recent findings that explain how MCs act as a link between the innate and adaptive immune response, all the way from sensing invading pathogens, dangerous situations, and allergens to orchestrating the final outcome of the immune reaction.

## 2. Innate MC Functions in Peripheral Tissues Fostering Adaptive Responses

MCs are tissue-resident myeloid cells, populating, at a high density, tissues lining the interface to the environment, such as skin, lung, and intestinal epithelium, and are also found in lower cell numbers in organ-defining barriers of the lymph nodes (LN), spleen, kidney, bone marrow (BM), and brain [3,10]. Due to their strategic positioning, MCs critically contribute to the first line of host defense against invading pathogens [4,11,12,13]. MCs are equipped with a wide array of pattern recognition receptors to identify invading pathogens, including Toll-like receptors (TLR), Fc receptors, and complement receptors [4,5,6,7,11,12]. Importantly, MCs have also been reported as sensors of cell stress and tissue damage, through alarmin and purinergic receptors [4,13,14,15,16]. Finally, MCs can be activated or modulated by binding cytokines, growth factors like stem cell factor (SCF), chemokines, and neuropeptides [14,15,16].

A unique characteristic of MCs is the high number of intracellular secretory granules, which in turn each contain a plethora of preformed mediators, such as histamine, proteases, cytokines, and chemokines [8]. An MC granule consists of a proteoglycan scaffold, in which the mediators are embedded based on electrostatic interactions [8,17]. Connective tissue-type murine MCs utilize heparin as the dominant proteoglycan, which allows for the detection of MC granules by metachromatic staining with Giemsa or Toluidine-blue, as well as by fluorochrome-conjugated avidin. In comparison, mucosal-type MCs in the lung and intestinal epithelial layers contain chondroitin sulfate-based granules [8,17]. Upon IgE/FcεRI crosslinking by a specific antigen or other stimuli, MCs release these secretory granules within only seconds to minutes, in a process called degranulation [1,4].

Because they can immediately degranulate, MCs respond to invading pathogens or cell stress faster than other tissue-resident immune cells and therefore, in many cases, are the initiators of immune responses. Whenever an inflammatory insult is causing MC degranulation, the immediate release of histamine triggers vascular responses, in particular vasodilatation and vessel permeabilization, within only minutes, finally leading to tissue edema [12,18,19,20,21,22]. The effect of histamine on endothelial cell activation and vascular barrier disintegration is potentiated by the MC release of tumor necrosis factor (TNF) [23,24,25] and proteases [26,27,28,29], as well as the rapid production of lipid mediators [30,31]. Complementing the vascular effects, MCs are also critical initiators of neutrophil recruitment, for example, during sepsis and peritonitis [26,32,33,34,35], upon lipopolysaccharide (LPS)-induced lung inflammation [36], as well as at sites of skin inflammation [18,37,38,39] and bone fracture [40] and in areas of arteriogenesis [41] and atherosclerotic plaque progression [42,43]. More specifically, MCs contribute to early neutrophil recruitment by the release of the neutrophil chemoattractants CXCL-1 (KC) and CXCL-2 (MIP-2), in addition to vascular effects [34,38,44,45]. Importantly, we have recently demonstrated in a model of contact hypersensitivity (CHS) that MCs degranulate directionally into the blood stream and thereby infuse TNF that primes circulating neutrophils for efficient extravasation [46]. Beside their boosting effect on neutrophil influx, MCs were also reported to enhance neutrophil effector functions [47,48]. In addition, MCs are a potent source of eosinophil-attracting chemokines (eotaxins) and, via histamine, inducers of eotaxin release by endothelial cells. Thereby, MCs are key drivers of eosinophil recruitment and have been shown to interact with eosinophils [49,50,51].

The MC-mediated vessel permeability and subsequent edema formation further support the recruitment of adaptive immune effector cells to the site of infection or inflammation. Indeed, by blocking the activity of MC-released histamine on the vasculature, subsequent T-cell-driven adaptive immune responses were severely impaired [18]. Furthermore, the edema-related relaxation of connective tissue is important for dendritic cell (DC) motility at the site of infection/inflammation and their subsequent migration toward the draining LNs (DLNs), in order to induce antigen-specific immune responses. Moreover, Weber et al. showed that, in CHS, (MC-initiated) neutrophil influx is required for an efficient activation and migration of DCs and hapten-specific T-cell priming, and consequently, for sensitizing efficiency of the hapten [37]. Neutrophil-released mediators with alarmin activity promote DC recruitment to sites of inflammation/infection and their maturation, thereby augmenting innate and adaptive immunity (reviewed in [52]). In contrast, neutrophil extracellular traps (NETs) and cathelicidins can downregulate LPS-induced DC activation and the T-cell priming capacity, in part by neutralizing LPS [53,54,55,56].

## 3. MC Functions in LN Conditioning and Hypertrophy

**Figure 1:**
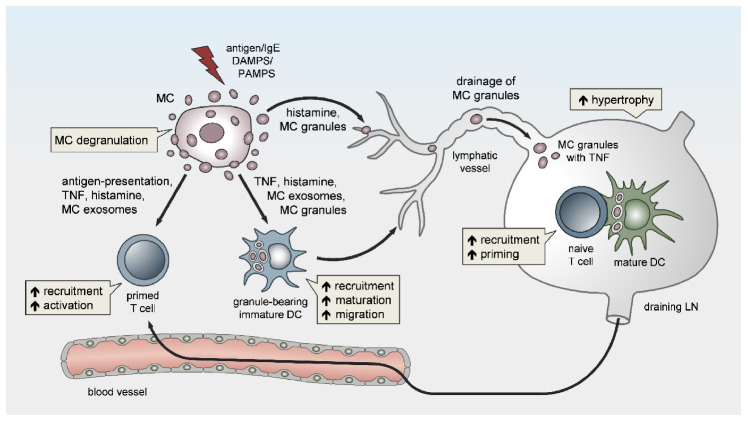
Peripheral mast cells (MCs) orchestrate the induction and amplitude of local innate responses and distant lymph node-borne adaptive immunity. The sensing of pathogens or danger-associated patterns by MCs or MC activation by IgE crosslinking in the periphery may result in MC degranulation and/or the de novo synthesis of pro-inflammatory mediators. Peripheral MCs exert remote effects on lymph node (LN) hypertrophy via histamine, TNF, and the drainage of intact MC secretory granules. The migration, maturation, and antigen-presenting capacity of dendritic cells (DCs) is promoted by MC soluble mediators, secretory granules, and exosomes, thereby facilitating T-cell expansion in draining LNs (DLNs). Finally, MCs enhance the homing of effector T cells to peripheral sites of inflammation/infection and may contribute to effector T-cell activation.

Besides local vasoactivation and edema formation, peripheral MCs support APC and lymphocyte influx in DLNs by exerting remote effects (Figure 1). Increased TNF levels have been detected in prenodal lymph and DLNs [57,58,59], as early as one hour after peripheral MC activation. Indeed, McLachlan et al. showed that, upon intradermal bacterial challenge, peripheral MC-derived TNF is the main driver of DLN hypertrophy and the recruitment of circulating T cells. In addition, MCs play a pivotal role in TNF-independent, but complement-regulated, LN hypertrophy and Langerhans cell mobilization, following intradermal peptidoglycan injection [56]. Moreover, *Anopheles mosquito* bite-induced dermal MC degranulation was not only shown to lead to local inflammation and neutrophil influx, but also to be required for T-cell and DC recruitment to the DLN, which is a prerequisite for T- and B-cell priming [60]. The mechanisms that underlie peripheral MC long-distance effects on DLNs and facilitate LN hypertrophy and circulating lymphocyte influx have barely been examined, but might be related to MC mediator drainage. Gashev and colleagues showed that, in rats, MCs reside close to mesenteric lymphatic vessels (MLVs) and direct the recruitment of MHC class II-positive cells [61,62]. The histamine release of perilymphatic MCs impacts the lymphatic microenvironment in an NFκB-dependent manner [63,64]. Importantly, the perilymphatic mesenteric MCs directly regulate themselves via histamine receptors in an autocrine loop, which is essential for acute inflammation-induced trafficking of MHC class II-expressing leukocytes [65]. Given the significant distance between the inflamed peripheral site and the DLN, it is still unclear how peripheral MC-derived cytokines, such as TNF, can reach the LN without being degraded or diluted to ineffective concentrations, particularly considering the short half-life period of TNF in vivo [66]. The remote effect of MC-derived TNF may be explained by its storage in the proteoglycan-backbone of the secretory granules. Importantly, we and others were able to visualize in vivo that the secretory granules are released by peripheral MCs in an intact and stable form [8,67,68]. Mediators such as histamine that are not highly charged rapidly diffuse from the proteoglycan matrix upon MC granule secretion to the extracellular fluid. In contrast, other mediators, such as MC proteases and TNF, are released slowly and sequentially from the secreted granules, which may enhance their activity and prolong their presence in the extracellular tissue [68,69,70]. Kunder et al. reported that, upon the topical application of phorbol-acetate-myristate (PMA), resulting in peripheral MC degranulation, some of the MC granules can enter the lymphatics and drain to local LNs, while no degranulation of LN-resident MCs was detected [68]. Furthermore, the authors demonstrated that the drained granules, carrying TNF, could efficiently elicit profound LN hypertrophy (Figure 1). Due to this adjuvant effect of MC granules, the same group modeled synthetic carbohydrate-backbone particles with encapsulated inflammatory mediators and showed their efficiency in enhancing adaptive immune responses upon influenza virus hemagglutinin vaccination [71].

## 4. MCs Affect Adaptive Immunity via the Modulation of Dendritic Cells

Beside the effect on LN conditioning and hypertrophy, MCs are indirectly implicated in LN-borne adaptive immune responses via the modulation of DC functions (Figure 1). In peripheral tissues, and particularly those lining the interface to the environment such as the skin, MCs reside in a dense network of tissue-resident innate immune cells and are involved in a variety of intercellular interactions [72,73]. We have previously shown that MCs and macrophages (Mph) cooperate in initiating the recruitment of neutrophils in a model of LPS-induced peritonitis [34]. However, despite their close proximity, the interaction between MCs and Mph and its impact on the recruitment and activity of effector T cells remains elusive. Several studies have reported intense communication between MCs and DCs and the MC-driven modulation of DC migration, maturation, and function, thereby linking MCs to adaptive responses [73,74]. On one hand, peripheral MC activation is critical for the recruitment of additional DCs to sites of bacterial infection and protective immunity [75]. On the other hand, MCs promote DC migration from the skin to the DLN after IgE-mediated activation [76,77], and in response to bacteria [75] or bacterial products [59,78].

**Figure 2:**
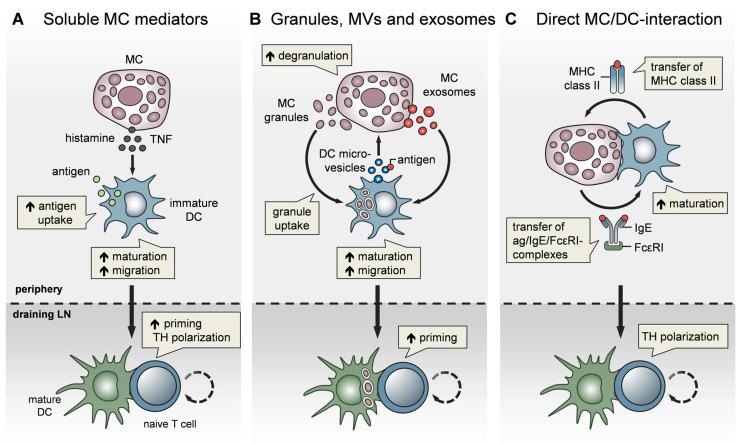
MCs impact T-cell activation by modulating DC functionality. MCs communicate with DCs in three different modes. (**A**) Soluble MC mediators, in particular histamine and TNF, promote the migration, maturation, and antigen-presenting capacity of DCs, thereby enhancing T-cell priming and fine-tuning T_H_ cell polarization. (**B**) MC exosomes and intact MC secretory granules, engulfed by DCs upon MC degranulation, facilitate DC migration and maturation, and consequently, boost T-cell priming. In turn, DCs relay antigen to MCs via extracellular microvesicles and thereby induce MC degranulation (**C**) MCs and DCs undergo dynamic physical interactions and synapse formation allowing bidirectional exchange. MCs transfer endocytosed antigen-IgE-FcεRI complexes to DCs, facilitating the activation of allergen-specific T cells. In turn, MCs are “cross-dressed” by DCs with MHCII complexes, thereby enabling the activation of effector T cells by MCs with antigen processed by DCs.

In terms of CHS, in a mouse model of T-cell-driven disease allergic contact dermatitis, we found that, upon hapten sensitization, MCs promote DC migration to skin-DLNs and DC maturation, and thereby critically enhance T-cell expansion [18]. Consequently, the expansion of both CD4^+^ and CD8^+^ T cells in skin-DLNs, and the T-cell-triggered adaptive skin inflammation upon hapten challenge, were markedly reduced in the absence of MCs [18]. In particular, peripheral TNF release by MCs is required for the efficient initiation of skin and airway DC migration to DLNs [78,79,80] (Figure 2A). Using MC-specific TNF knockout, we could show in vivo that MC-derived TNF predominantly targets cDC1 migration and priming capacity upon hapten sensitization, thereby promoting CD8^+^ effector T-cell responses [80].

In addition to their effect on DC migration, MCs have been reported to enhance DC maturation, antigen processing, and T-cell priming capacities. Specifically, histamine promotes DC maturation [81], antigen uptake, and cross-presentation [82] and regulates the DC cytokine response, thereby polarizing T cells toward a Th2 phenotype [83]. In line with this, IgE-stimulated MCs control the Th1/Th2 balance by promoting Th2-generating DCs [84,85] (Figure 2A). Importantly, MCs exert effects on DC functionality not only by soluble mediators, but also via the secretory MC granules, MC-derived exosomes, and physical contact (Figure 2). By means of MC granule staining in vivo, directly inside the MCs, and intravital 2-photon-microscopy, we could monitor MC degranulation and track the fate of MC granules after their exocytosis. We found that, upon skin inflammation, dermal DCs accumulate at the site of MC degranulation, engulf the intact MC granules, and actively shuttle them to skin-DLNs [67]. This MC granule uptake facilitates DC migration and maturation, and boosts their T-cell priming capacity (Figure 2B). The cDC1 subpopulation was the most efficient in MC granule uptake in a partially TNF-dependent manner. Importantly, the intradermal (i.d.) injection of MC granules into MC-deficient mice was able to induce a profound expansion of T cells, indicating their adjuvant effect. Extending the finding of Kunder et al. [68], we provided evidence that MC degranulation in the periphery may exert long-distance effects on LN-borne adaptive T-cell responses in two ways: (a) The trafficking of MC granules via lymphatic vessels towards the DLNs, and (b) the active shuttling of MC granules by DCs along with DC-modulating effects [67].

In addition to MC granules, MC-derived exosomes offer an additional mechanism for intercellular communication, by having a greater stability in the interstitial space compared to soluble mediators [72,86] and being able to promote DC maturation and the antigen-presenting capacity [87,88] (Figure 2B). Importantly, DCs may also communicate with MCs and vice versa, via extracellular microvesicles. Choi et al. recently demonstrated that CD301b^+^ perivascular DCs sample the blood and relay blood antigens to neighboring perivascular MCs, which can subsequently result in antigen/IgE-crosslinking-induced MC degranulation and an anaphylactic response [89].

Confirming our findings on CHS, Otsuka et al. showed impaired skin DC maturation and migration in the absence of MCs and highlighted the relevance of a direct interaction between DCs and MCs, leading to an upregulation of membrane-bound TNF by MCs [90]. Given the close proximity of MCs and DCs in peripheral tissues, especially in the skin, a physical cell-to-cell interaction was considered likely and studied in several in vitro studies (Figure 2C). Non-activated peritoneal MCs, resembling connective tissue-type skin MCs, underwent direct crosstalk with immature DCs, inducing DC maturation and CD4^+^ T-cell polarization toward Th1 and Th17 responses [91]. Upon FcεRI crosslinking, MCs have been shown to form immunological synapses with DCs, enabling the transfer of endocytosed antigens from MCs to DCs to activate T cells [92] (Figure 2C). However, there is still little in vivo evidence for a functional relevance of the MC/DC cell-to-cell interaction. In a recent study, using intravital 2-photon-microscopy of MC/DC double reporter mice, we demonstrated in vivo, for the first time, that upon skin inflammation, MCs and DCs rapidly undergo a highly dynamic interaction, which evolves to long-term synapse formation [93]. This MC/DC communication culminates in a protein exchange from DCs to MCs, including MHC class II complexes. Intriguingly, the DC “cross-dressing” of MCs with functionally active MHC class II complexes equipped the MCs with an antigen-presenting capacity, which subsequently enhanced T-cell-driven skin inflammation (Figure 2C). This MC bestowal with antigen-presenting capacity, particularly antigens that have been engulfed and processed by DCs before leaving the peripheral tissue towards the DLN, suggests a role for MCs in activating effector T cells that enter the peripheral tissue [93].

## 5. Direct Role for MCs in T-Cell Activation

In addition to the modulation of the APC function in the periphery, MCs have been reported to directly impact T-cell activation. Here, MCs may exert promoting effects by two modes of action: Either by direct antigen-presentation or by modulating T-cell expansion, differentiation, and polarization via soluble mediators.

### 5.1. Antigen-Presenting Capacity of MCs

The capacity of MCs to directly present antigen to T cells has been speculated by several reports based on the finding that MCs may express MHC class II complexes under certain conditions. An early study showed a selective ability of BM-derived mouse MCs (BMMCs) to present exogenous antigens that is supported by granulocyte macrophage colony stimulating factor (GM-CSF) [94]. Kambayashi et al. demonstrated the MHC class II expression and antigen-presenting capacity of BMMCs and splenic mouse MCs, in response to LPS and IFN-γ in vitro and upon inflammatory insult in vivo [95]. This finding was supported by Gaudenzio et al., who showed the expression of MHC class II and costimulatory molecules (CD80 and CD86) on mature peritoneal mouse MCs stimulated with IFN-γ and IL-4 [96]. Interestingly, the antigen-presenting capacity relied on direct MC/T-cell crosstalk, where CD4^+^ T cells formed immunological synapses and polarized their secretory machinery toward the antigen-loaded MCs [96]. In BMMCs, the MHC class II expression is induced by Notch ligand Delta-like 1 (Dll1)/Notch signaling through activation of the class II transactivator (CIITA) [97,98]. Furthermore, IgE/antigen-stimulated BMMCs enhance T-cell activation by the expression of various costimulatory molecules, including ICOSL, PD-L1, PD-L2, Ox40L, Fas, and 4-1BB, and a TNF-mediated increase of the surface expression of the respective counter-receptors on T cells [99].

In line with this, the expression of HLA-DR and activation of antigen-specific T cells were confirmed for human MCs that were stimulated with IFN-γ or by FcεRI crosslinking [100,101]. Here, the direct crosstalk between tonsillar human MCs and CD4^+^ T cells seemed to involve costimulation via Ox40L/Ox40 [101].

**Figure 3:**
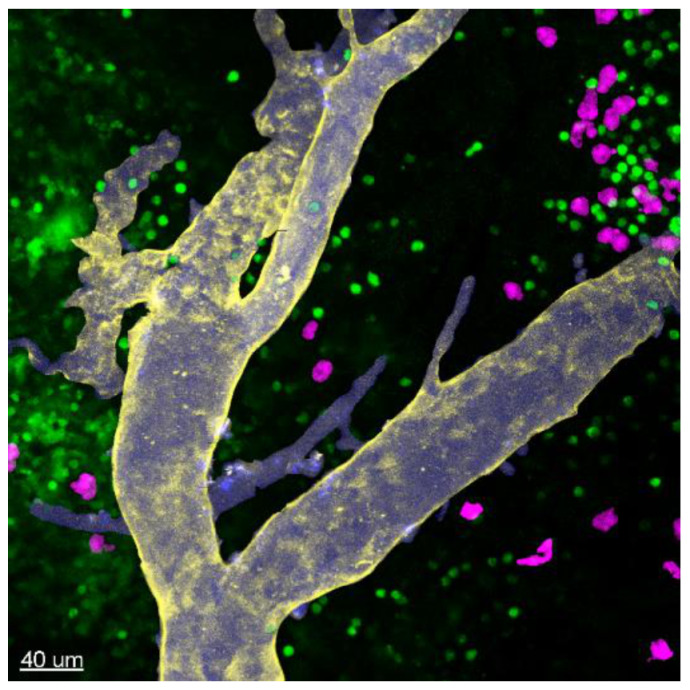
MC accumulation in LN T-cell zones upon immunization. Two-photon-microscopy of MC/T-cell double reporter mice revealed the accumulation of MCs in the T-cell zone and colocalization with T cells in inguinal LNs six days after intradermal immunization with collagen/CFA. Green: T cells; purple: MCs; blue: vessel tracer Angiospark 750; and yellow: anti-CD31Ab (quantification and more detailed information in [102]).

**Figure 4:**
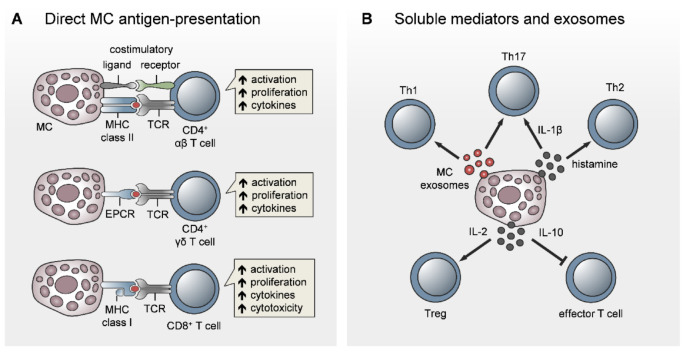
Direct role for MCs in the activation and modulation of T-cell responses. There is increasing evidence that MCs contribute to T-cell activation, not only by direct antigen-presenting potential, but also by modifying the outcome of the T-cell response. (**A**) Direct MC/T-cell interaction and synapse formation and the antigen-presenting capacity of MCs have been shown for CD4^+^ αβ T cells, γδ T cells, and CD8^+^ T cells. (**B**) Beyond direct activation, MCs can modulate T-cell activation by exosomes and soluble mediators. Here, MCs skew T-cell polarization towards Th1, Th17, or Th2, depending on the mode of MC stimulation. In addition, MCs provide anti-inflammatory effects by promoting T_reg_ activation via IL-2 or by inhibiting conventional T-cell activation via IL-10 in a T_reg_-independent way.

Despite robust in vitro evidence for the antigen-presenting potential of MCs, MC interactions with naïve T cells in LNs or with effector T cells infiltrating peripheral sites of inflammation/infection have barely been explored in vivo. In collagen-induced arthritis (CIA), MC-depleted mice showed reduced joint inflammation due to impaired T-cell expansion and T-cell cytokine response upon i.d. collagen/complete Freund’s adjuvant (CFA) immunization [102,103]. When exploring the underlying mechanism, we only found a few MCs in the subcapsular regions of inguinal LNs under physiological conditions. MCs were accumulated in the LN T-cell zones, but only later on (at day 6 after immunization), thereby succeeding effector T-cell expansion and egress from the LNs [102] (Figure 3). This finding let us speculate once again that MC effects on T-cell priming may be linked to DC modulation during immunization. Consequently, the MC antigen-presenting function may be important for effector T-cell activation in the periphery rather than naïve T-cell priming in lymphoid organs. Indeed, the in vitro studies by Gaudenzio et al. and Kambayashi et al. showed that MC antigen-presentation preferentially induced the expansion of antigen-specific effector T cells and regulatory T cells over naïve T cells [95,96] (Figure 4A). Our hypothesis of MC antigen-presentation in peripheral tissues is supported by a recent study by antigens under hyperlipidemic conditions [104]. Importantly, Kritikou et al. showing an increased MHC class II expression and in vivo capacity to present, the authors identified HLA-DR-expressing MCs in human atherosclerotic plaques, in line with reduced aortic CD4^+^ T-cell numbers and proliferation in MC-deficient mice [104]. In a non-conventional mode of antigen-presentation, MCs induce γδ T-cell activation and proliferation in dengue virus-infected peripheral tissue, due to immune synapse formation mediated by the T-cell receptor and the endothelial cell protein C receptor (EPCR) [105] (Figure 4A).

In addition to MHC class II-dependent activation of CD4^+^ T cells, MCs have been shown to induce CD8^+^ T-cell activation and proliferation and to promote CD8^+^ T-cell cytokine release and cytotoxicity in a direct cell contact and MHC class I-dependent manner [106]. This finding was confirmed in vivo, since the adoptive transfer of antigen-pulsed MCs induced CD8^+^ T-cell priming in experimental autoimmune encephalomyelitis (EAE) (Figure 4A).

### 5.2. MC Modulation of T-Cell Priming, Differentiation, and Polarization

In addition to the potential MC function as APCs, several studies have reported MC-driven modulatory effects on T-cell priming, differentiation, and polarization (Figure 4B). However, most of the work has been conducted in vitro using immature BMMCs, which complicates interpretation of the functional relevance under disease conditions. For example, the co-activation of T cells in the presence of IgE/antigen-activated BMMCs skewed the T-cell response towards IL-4producing Th2 cells [107]. In particular, MC-derived histamine may regulate the Th1/Th2 balance by the differential expression of H1 and H2 histamine receptors [108]. In contrast, Liu et al. recently reported a role for the MC-derived mouse mast cell-protease 6 (Mcpt6) in counter-regulating Th2 polarization and cytokine release by increasing Bcl-6 in Th2 cells, which subsequently inhibited GATA-3 [109]. Human MCs have been demonstrated to enhance the Th17 fraction within the memory CD4^+^ T-cell population by inflammasome-independent IL-1β release [110].

In addition to soluble MC mediators, B- and T-cell activation is regulated by the secretion of MC exosomes harboring immunologically relevant molecules, such as MHC class II, CD86, LFA-1, and ICAM-1 [69,88,111]. Purified MC exosomes have been demonstrated to induce blast formation, T-cell proliferation, and IL-2 and IFN-γ production, while being inefficient in the induction of IL-4 [111] (Figure 4B). IL-2 production by BMMCs, in response to concomitant IL-33 signaling and FcεRI activation, resulted in the expansion of regulatory T cells in vitro. Salamon et al. demonstrated elevated IL-33 levels and increased numbers of IL-2-expressing MCs in human skin with chronic inflammation and in mouse ear skin upon allergic dermatitis and concluded that MC-derived IL-2 has a role in Treg stimulation and the suppression of allergic dermatitis [112]. Independent of regulatory T cells, MCs have been reported to suppress graft-versus-host disease by decreasing conventional T-cell proliferation via release of the anti-inflammatory cytokine IL-10 [113]. MC-delivered exosomes were further involved in a recently described non-conventional mechanism, supporting the Th17 response in the chronic inflammatory skin disease psoriasis [114]. In detail, the cytoplasmic phospholipase A2 (PLA2G4D) is expressed by MCs upon psoriasis and transferred within exosomes to neighboring CD1a-expressing Langerhans cells. The resulting presentation of neolipid antigens to lipid-specific CD1a-binding T cells induced the production of IL-22 and IL-17A, driving skin inflammation [114].

## 6. MC Functions in B-Cell Activation

In contrast to the MC-T-cell axis, there is much less knowledge regarding MC functions in regulating B-cell numbers, activation, or antibody responses. Due to the accumulation of MCs and MC-driven effects in B cell-mediated inflammatory disorders, including rheumatoid arthritis, a direct modulation of B cells by MCs was hypothesized. Moreover, the high levels of IL-6 released by MCs suggest MC–B-cell communication, for example, in pulmonary hypertension [115]. In MC-deficient Kit mutant mice, an impaired protective humoral response to *Escherichia coli* was observed, which led to the suggestion of pharmacological MC activation as a new adjuvant principle in vaccination [72,116,117]. However, a non-redundant role for MCs in antibody production could not be confirmed in Kit-independent novel mouse models of MC-deficiency [118].

In vitro, naïve, sensitized, and activated MCs were shown to promote the proliferation of naïve and B-cell receptor-activated B cells [119,120,121], as well as both follicular and marginal zone B cells [118]. As indicated by the secretion of IgM and IgG by IgM^+^ B cells, MCs can induce class switch recombination [118]. Pucillo and colleagues further reported that the CD40/CD40L-mediated MC/B-cell contact, together with IL-6 secretion by MCs, differentiates B cells to CD138^+^ plasma cells and leads to IgA secretion [117]. The same group demonstrated the existence of MC/B-cell crosstalk in the inflamed colon of inflammatory bowel disease (IBD) patients and, by using MC-depleted mice, confirmed, in vivo, a role for MCs in the control of B-cell distribution in the gut, as well as in increased IgA production upon dextran sulfate sodium (DSS)-colitis [122]. In vitro, MCs regulated splenic B cells, while peritoneal B cells were unresponsive but skewed the MCs to increased IL33 receptor expression and TNF production [119]. The synthesis of IgE by B cells was found to be enhanced by adenosine-activated human MCs via IL-4 and IL-13 production, which is a process that might be implicated in asthma-associated amplification of allergic inflammatory responses [123]. In contrast, Kim et al. recently described an immunoregulatory function of MCs in the control of severe CHS. Here, the MC production of IL-5 maintains the population of IL-10^+^ regulatory B cells in peripheral tissues, which in turn suppress the activation of IL-13 producing type 2 innate lymphoid cells in an IL-10-dependent manner [124].

## 7. MCs Orchestrate Effector Cell Recruitment to Inflamed Tissues

While MCs are known for their role in neutrophil and eosinophil recruitment, only a few reports have addressed, until now, the impact of MCs on effector T-cell recruitment to peripheral tissues. This limited knowledge may arise from the restriction of cell dynamic and recruitment studies to in vivo mouse model investigations. On the other hand, MC effects on T-cell recruitment are hard to discriminate from effects on T-cell expansion and activation. Hence, the reduction of CD4^+^ and CD8^+^ T-cell numbers, infiltrating the ear skin upon allergic contact dermatitis, which we have observed in the absence of MCs, may include MC effects on the recruitment process itself, in addition to effects on LN-borne T-cell expansion [18,80]. Determining specific MC functions in effector T-cell recruitment requires the uncoupling of T-cell priming from T-cell extravasation, for example, by site-specific MC depletion (possible in novel mouse models of diphtheria toxin-induced MC depletion [18,90,125]), or by adoptive transfer approaches. However, vessel endothelium activation by MCs, in line with their capacity to produce the T-cell chemoattractants CCL2 (MCP-1), CCL5 (RANTES), and CXCL10 (IP-10), indicates a contribution to the recruitment of effector T cells, once they are expanded in lymphoid organs, to the peripheral site of inflammation or infection [126,127,128,129]. MCs have been suggested as attractors of CD8^+^ effector T cells in two studies. In an early in vitro report, activated MCs induced the chemotaxis of effector, but not central memory, CD8^+^ T cells through the production of leukotriene B4 (LTB4) [130]. In vivo, Ebert et al. showed that systemic infection with cytomegalovirus (CMV) induced MC degranulation selectively in infected MCs, thereby eliciting a wave of CCL5 [131]. In MC-deficient mice, CD8^+^ T cells were recruited less efficiently to the lungs, which correlated with enhanced viral replication and delayed virus clearance [128].

## 8. The Adjuvant Effect of MC Activators in Vaccination

Given the fact that MCs promote LN hypertrophy and T-cell expansion and even contribute to B-cell responses and antibody production, beneficial MC functions during vaccination have been hypothesized. In particular, the groups of Abraham and Staats followed their own observations [58,68,75], with the idea of using adjuvants that target MCs specifically, termed MC activators, during vaccination against protein antigens. Firstly, the MC-activating compound 48/80 (c48/80), which is a calcium ionophore, has been proven as an effective and safe adjuvant for immunization with several protein antigens, such as botulinum neurotoxin A [132], the *Bacillus anthracis* protective antigen [116,117,133], the influenza H1N1 recombinant hemagglutinin protein [134], and the hepatitis B surface antigen [135], resulting in enhanced antibody production. Since MCs are located in mucosal surfaces at a high density, c48/80 was also efficiently used as adjuvant in powder vaccines for intranasal application [132,133,134,136]. In addition, the incorporation of c48/80 into chitosan nanoparticles as a delivery system has been demonstrated to further enhance mucosal immunity [133,135,137]. C48/80 can also efficiently enhance the synthesis of IgE and IgG upon the intranasal application of ovalbumin [138]. In contrast, c48/80 displayed no adjuvant activity for immunization with UV-attenuated *Toxoplasma gondii* [139]. Surprisingly, a promoting effect of MCs on antibody production upon protein vaccination could not be confirmed in a transgenic model of MC deficiency independent of kit mutations, which may be attributable to the fact that only connective tissue-type, but not mucosal, MCs were depleted [118].

Some MC activators target the MC-specific mas-related G-protein coupled receptor MrgprX2 (or its murine orthologue MrgprB2). The core advantage is the specificity to MCs, thereby avoiding unspecific uncontrollable immune reactions, such as the ones caused by CpG and other Toll-like receptor-targeting adjuvants, or alum. Importantly, McNeil et al. demonstrated that the mouse orthologue MrgprB2 is activated by basic secretagogues, such as c48/80 and mastoparan, by neuropeptides such as substance P, but also by a number of FDA-approved peptidergic drugs, such as icatibant [140,141]. The binding to MrgprB2 may be responsible for the induction of pseudo-allergic anaphylactic responses and injection-site inflammation by some peptidergic drugs. Moreover, the authors identified a common chemical motif of many small-molecule drugs associated with pseudo-allergic reactions that may help predict the side effects of other compounds in the future [141]. Although c48/80 acts on MrgprB2/MrgprX2, Schubert et al. provided evidence that the adjuvant effects of c48/80 in intradermal or mucosal immunization may also be independent of MC activation. Other MrgprB2/MrgprX2 binding peptides, such as mastoparan, which is a wasp venom component, may be more specific [140]. Hence, Abraham and colleagues could show that (i) mastoparan-induced MC activation promotes the clearance of bacterial infection and protects against reinfection [142], (ii) a mastoparan derivative has broad-spectrum antiviral activity [143], and (iii) the MC-activating oligopeptide mastoparan-7 (M7) can induce effective humoral immunity upon cocaine vaccination [144]. In humans, the antimicrobial peptide LL-37, which is a member of the cathelicidin family, has been demonstrated to induce MC activation by binding to MrgprX2 [145,146]. Kim et al. found that, when used in a mouse model, LL-37 has potential as an oral mucosal adjuvant, by promoting T-cell-mediated and Th17-skewed antigen-specific mucosal and systemic immunity [147]. Other compounds that induce MC degranulation and provide potent adjuvant effects, upon intranasal protein immunization, include the bee venom component melittin [148,149], and the antibiotic cyclic lipopeptides polymyxin B [150,151] and surfactin [152]. MCs also contribute to the potential adjuvant effect of interleukin-1 family cytokines, upon intranasal immunization with recombinant influenza virus hemagglutinin [153]. Finally, Abraham and colleagues showed that chitosan-based synthetic MC granules provide adjuvant effects that promote and polarize LN-borne adaptive immune responses [71].

## 9. Concluding Remarks

MC research of the last two decades has provided increasing evidence that MCs critically contribute to innate host defense and adaptive immunity. In peripheral tissues, MCs sense pathogens and danger-associated patterns and initiate local innate responses. Beyond that, MCs affect the onset, kinetics, and amplitude of adaptive immunity by (at least) four modes of action: (a) Remote effects initiating draining LN hypertrophy; (b) Promoting DC migration and functionality; (c) Inducing or modulating T-cell activation and polarization; and finally, (d) Orchestrating the homing of effector T cells to the site of inflammation or infection. Despite our current advances, future work is required to substantiate recent findings and indications with in vivo evidence, particularly using novel mouse models of MC deficiency or MC-specific gene inactivation, independent of Kit mutations. Moreover, due to their immobility, the capacity of MCs to link innate sensing to the induction and fine-tuning of adaptive immune responses relies on cellular communication. Therefore, understanding MC communication with neighboring tissue-resident immune cells and infiltrating effector cells should be the principal focus, in order to reveal future therapeutic targets to either intentionally boost or dampen adaptive immunity.
